# Dental Complications in Antithrombotic Patients: Evidence From a Nationwide Cohort and a Single-Institution Dataset

**DOI:** 10.1016/j.identj.2026.109649

**Published:** 2026-06-01

**Authors:** Jaeyeon Kim, Joon-Ho Yoon, Jisun Huh, Geun U. Park, Seo-Yeon Gwak, Yiseul Choi, Jun-Young Kim, Chi Young Shim, Wonse Park

**Affiliations:** a Department of Advanced General Dentistry, College of Dentistry, Yonsei University, Seoul, South Korea; b Department of Prosthodontics, National Health Insurance Service – Ilsan Hospital, Goyang, South Korea; c Department of Dental Education, College of Dentistry, Yonsei University, Seoul, South Korea; d GN, Seoul, South Korea; e Division of Cardiology, Korea University Guro Hospital, Korea University College of Medicine, Seoul, South Korea; f Department of Oral and Maxillofacial Surgery, College of Dentistry, Yonsei University, Seoul, South Korea; g Yonsei Institute for Digital Health, Yonsei University, Seoul, South Korea; h Division of Cardiology, Severance Cardiovascular Hospital, Yonsei University College of Medicine, Seoul, South Korea; i Oral Science Research Center, College of Dentistry, Yonsei University, Seoul, South Korea

**Keywords:** Dentistry, Electronic Health Records, Insurance Claim Review, Postoperative Complications, Hemorrhage, Thromboembolism

## Abstract

**Introduction and aims:**

This study aimed to evaluate the incidence and risk factors of postoperative bleeding and systemic complications occurring following dental procedures in patients undergoing antithrombotic therapy, using a nationwide cohort and single-institution clinical data.

**Methods:**

This retrospective cohort study analysed data from the (1) National Health Insurance Service–National Sample Cohort, representing 2.2% of the Korean population and (2) Severance Clinical Research Analysis Portal 2.0 at Yonsei University Dental Hospital. The study included adults aged ≥30 years who were diagnosed with cardiovascular disease and received dental treatment from 2015 to 2019.

**Results:**

Among 714,397 dental treatment cases in the National Health Insurance Service–National Sample Cohort, systemic bleeding and thromboembolic complications requiring hospitalization or resulting in death occurred in 0.30% and 0.06% of cases following dental treatment. In the single-institution dataset (n = 1,878), postoperative bleeding occurred in 2.18% of cases. Hypertension and diabetes mellitus were associated with systemic bleeding. Patients who received thrombolysis had a higher risk of thromboembolic complications, and vitamin K antagonists with heparin bridging were significantly associated with postoperative bleeding. Tooth extraction and implant surgery with bone grafting were associated with higher risks of postoperative bleeding, but implant placement without bone grafting was not significantly associated.

**Conclusion:**

VKAs with heparin bridging and implant surgery with bone grafting were associated with higher risks of postoperative bleeding and systemic complications. In contrast, the relatively low bleeding risk with single antiplatelet therapy and implant placement alone that dental procedures may be performed with an acceptable safety profile in patients receiving antithrombotic therapy.

**Clinical relevance:**

This study provides real-world evidence on dental and systemic complications following dental procedures in patients receiving antithrombotic therapy. Procedural invasiveness, antithrombotic management, and patient comorbidities were relevant factors for complication risk and may support risk stratification and procedural planning in dental practice.

## Introduction

Cardiovascular disease (CVD), a group of disorders affecting the heart and blood vessels, including coronary artery disease, cerebrovascular disease, and peripheral artery disease, is a major cause of mortality worldwide, accounting for 15.8% of all deaths in South Korea.^1^, ^2^, ^3^ Major risk factors include hypertension, diabetes, and dyslipidemia, thereby increasing the use of antithrombotic agents for primary and secondary prevention as populations age.^4^

Antithrombotic therapy includes antiplatelet and anticoagulant agents. Common antiplatelet drugs, such as aspirin, clopidogrel, prasugrel, and ticagrelor, are used as single antiplatelet therapy (SAPT) or dual antiplatelet therapy (DAPT), particularly after angioplasty or stent implantation.^5^^,^^6^ Oral anticoagulants include vitamin K antagonists (VKAs) and direct oral anticoagulants (DOACs). Although DOACs have replaced VKAs in many indications, VKAs remain the first-line therapy for patients with mechanical heart valves or valvular atrial fibrillation because of their proven efficacy and reversibility.^7^, ^8^, ^9^

Many studies have reported bleeding risks after dental treatment in patients undergoing antithrombotic therapy.^10^ Recent guidelines recommend continuing SAPT or VKAs (international normalized ratio [INR] <3.5) for most dental procedures without significant bleeding risk.^11^, ^12^, ^13^ However, current evidence remains limited regarding the assessment of postoperative bleeding and systemic complications associated with dental procedures.^14^ Furthermore, most previous studies lack detailed dental procedure classifications, limiting clinical applicability.^15^^,^^16^

Although SAPT has been associated with a relatively low risk of non-major bleeding, DAPT has a higher incidence of bleeding complications, including intracranial hemorrhage.^17^^,^^18^ In patients taking VKAs, the annual incidence of intracranial hemorrhage ranges from 0.3% to 0.6%;^19^ however, data regarding systemic complications in patients taking DOACs remain limited despite their increasing use.^20^ Therefore, sufficient numbers of patients are needed to compare systemic complications among different antithrombotic agents.

This retrospective cohort study aimed to evaluate the risk of postoperative bleeding and systemic complications after dental procedures in patients undergoing antithrombotic therapy, using the National Health Insurance Service Sample Cohort (NHIS-NSC) and Severance Clinical Research Analysis Portal (SCRAP 2.0).

## Materials and methods

### Study design and ethical approval

This study was approved by the Institutional Review Board of the Yonsei University Dental Hospital (single-institution study: IRB No. 2-2023-0067; NHIS cohort study: IRB No. 2-2022-0072 and NHIS Study No. NHIS-2024-2-040). All data were anonymized before analysis. The requirement for written informed consent was waived owing to the retrospective nature of the study. This study was conducted in accordance with the principles of the Declaration of Helsinki.

### Data source

This study used two data sources:

1. **National Health Insurance Service–National Sample Cohort (NHIS-NSC):** The NHIS is a mandatory nationwide healthcare system that records all in- and outpatient procedures and prescriptions in Korea. The NHIS-NSC is a population-based cohort that includes 2.2% of the Korean population selected through stratified random sampling. The dataset includes individual qualification and contribution databases, health insurance claims (payment specifications [20T], consultation statements [30T], diagnosis statements determined by the International Classification of Diseases, 10th Revision [ICD-10] [40T], and detailed statements about prescriptions [60T]), health checkup databases, and medical institution databases. NHIS data are widely used in health and policy research (https://nhiss.nhis.or.kr).
2. **Severance Clinical Research Analysis Portal (SCRAP 2.0):** SCRAP 2.0 is the clinical data platform of the Yonsei University Health System that includes electronic medical records (EMRs) and order communication systems, providing comprehensive clinical data, including diagnoses, surgeries, pathology results, imaging tests, prescriptions, clinical observations, and diagnostic tests.

Each dataset was analysed independently due to differences in data structure and variable availability. The results of each dataset were interpreted in a complementary manner, and consistent inclusion criteria, study durations, and definitions of key variables (e.g., cardiovascular disease, dental procedures, drug exposure) were applied to both datasets. However, complete standardization was not possible because the NHIS-NSC dataset is based on claims data, whereas SCRAP 2.0 provides detailed clinical data.

### Study population

**Fig. 1:**
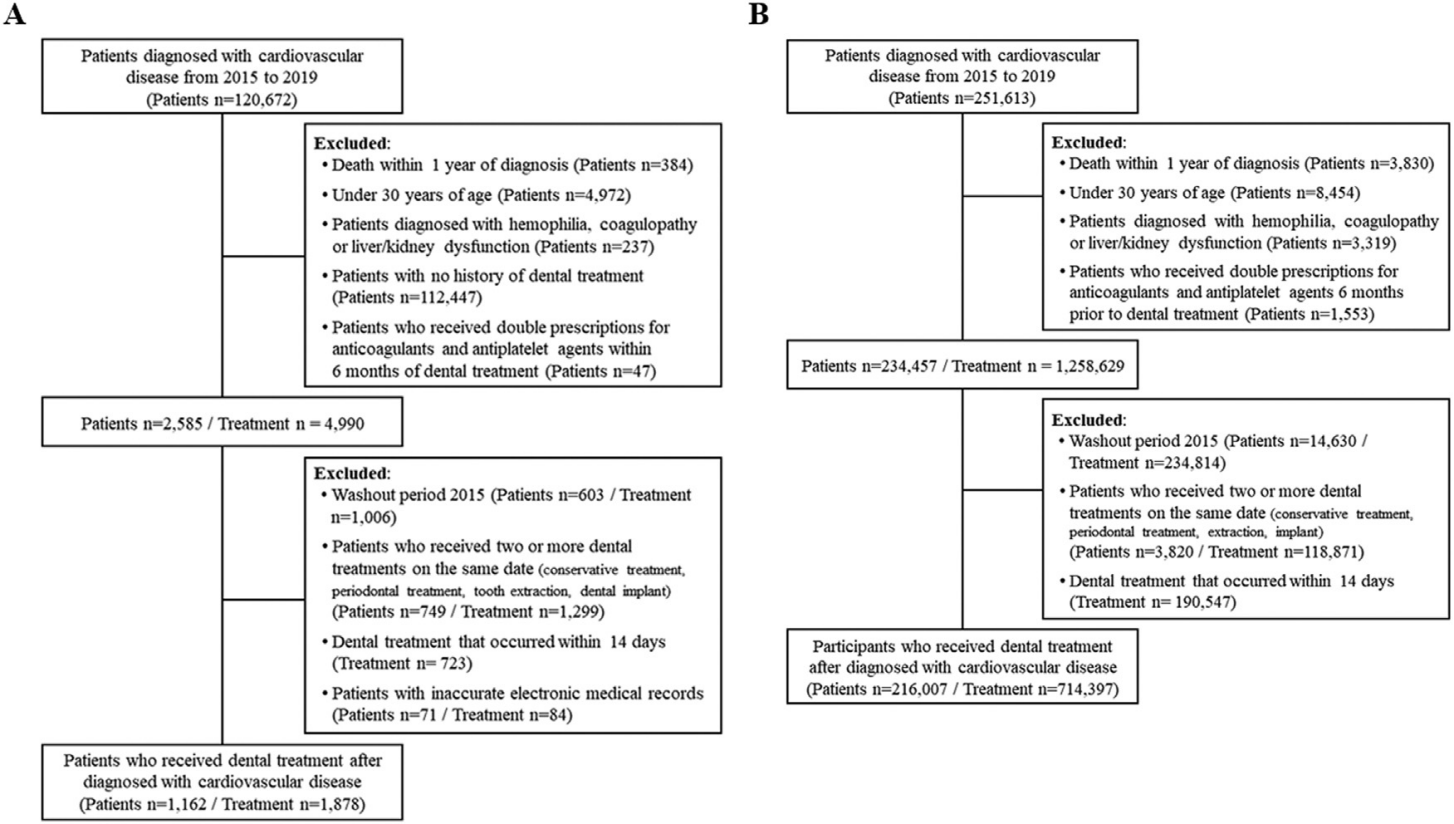
Flowchart of the inclusion and exclusion criteria for this study in each database. (A) Single-institution, (B) National Health Insurance Service Cohort Database.

Patients with CVD were identified based on ICD-10 codes recorded as the main or secondary diagnosis (Supplemental Table 1). The study period for both datasets was between January 2015 and December 2019, and the index date was defined as the initial diagnosis of CVD during this period. Adults aged ≥30 years who received dental treatment after CVD diagnosis were included. Patients who died within one year after diagnosis, those with hemophilia, coagulation factor deficiency, or hepatic or renal dysfunction, those prescribed with antiplatelet or anticoagulant agents within six months before dental treatment, those who underwent multiple dental procedures on the same date or within 14 days, and those with incomplete or inaccurate data (applicable only to single-institution studies) were excluded (Figure 1).

### Variables

Demographic variables included age (categorized into 30-39, 40-49, 50-59, 60-69, and ≥70 years) and sex. Medical variables were based on procedure codes for stent insertion, coronary artery bypass grafting (CABG), thrombolysis, heart valve surgery, and comorbidities, such as hypertension, diabetes, and dyslipidemia, identified using ICD-10 codes.

Medication variables were defined from the records of anticoagulant (VKAs and DOACs such as dabigatran, rivaroxaban, apixaban, and edoxaban) and antiplatelet (cyclooxygenase inhibitors, phosphodiesterase inhibitors, adenosine diphosphate inhibitors, 5HT2R antagonists, and DAPT using aspirin and clopidogrel) agents prescribed within six months before dental treatment. For the single-institution study, drug discontinuation status and dates were collected.

Dental treatment variables included conservative treatment (oral cavity preparation), periodontal treatment (scaling, curettage, root planing, and periodontal flap operation), tooth extraction (simple and surgical extractions, removal of fractured teeth, and hemisection), and implant-related surgeries (implant placement, removal, and bone grafting).

### Study endpoints

The definition of outcomes was determined based on the outcomes of interest. The NHIS-NSC dataset was used to evaluate systemic complications using a population-based cohort, and the SCRAP 2.0 dataset enabled the identification of clinically significant postoperative bleeding events through detailed EMRs, which are not reliably captured in claims data. Postoperative bleeding variables were evaluated based on the occurrence of bleeding reported by the patient or bleeding-related emergency room visits. Systemic complication evaluation variables were defined as hospitalization or death due to complications within 30 days of dental treatment. Systemic bleeding complications included clinically significant bleeding events such as intracranial hemorrhage, respiratory bleeding, hematuria, and anemia due to bleeding. Thromboembolic complications included stroke, arterial embolism, thrombosis, and other complications after acute myocardial infarction (Supplemental Table 2).

### Statistical analysis

All statistical analyses were conducted separately for each dataset. Categorical variables are presented as numbers and percentages, and continuous variables as means and standard deviations (SD). Chi-square test and independent Student’s t-test were used for categorical and continuous variables, respectively. Logistic regression analysis was conducted to evaluate the effects of demographic, medical, medication, and dental characteristics on the outcomes. All variables were entered into the multivariable model to adjust for confounders. Multicollinearity was assessed using variance inflation factors, and no significant multicollinearity was observed. Model fit was assessed using the Hosmer–Lemeshow test. Statistical significance was set at *P* < .05, and all analyses were conducted using SAS software (version 9.4, SAS Institute).

## Results

Between January 2015 and December 2019, 1,878 patients with CVD from Severance Hospital underwent dental treatment at Yonsei University Dental Hospital. Of these, 55.22% were male and most were in their 50s and 60s (Table 1). Antithrombotics were prescribed in 43.4% of cases, mainly antiplatelets, and the most common dental treatment was periodontal (54.37%). In the NHIS cohort (n = 714,397), 52.0% were men, and most were in their 50s to 60s. Antithrombotics were prescribed in 22.6% of cases, mostly antiplatelets (21.56%), and 0.99% received anticoagulants. The detailed characteristics are summarized in Supplemental Tables 3 to 6.

**Table 1:** Demographic and clinical characteristics in patients diagnosed with cardiovascular disease.

Characteristics	Single-institution dataset (n = 1,878)	NHIS-NSC dataset (n = 714,397)
	N (%)	N (%)
**Sex**		
Male	1,037 (55.22)	371,769 (52.04)
Female	841 (44.78)	342,628 (47.96)
Age		
30 to 39	81 (4.31)	28,044 (3.93)
40 to 49	243 (12.94)	92,355 (12.93)
50 to 59	483 (25.72)	211,413 (29.59)
60 to 69	591 (31.47)	218,929 (30.65)
≥70	480 (25.56)	163,656 (22.91)
**Comorbidities**		
Hypertension		
Yes	804 (42.81)	414,214 (57.98)
No	1,074 (57.19)	300,183 (42.02)
Diabetes mellitus		
Yes	827 (44.04)	278,305 (38.96)
No	1,051 (55.96)	436,092 (61.04)
Dyslipidemia		
Yes	931 (49.57)	467,768 (65.48)
No	947 (50.43)	246,629 (34.52)
**CVD surgery**		
Coronary stenting		
Yes	222 (11.82)	5,632 (0.79)
No	1,656 (88.18)	708,765 (99.21)
CABG		
Yes	17 (0.91)	194 (0.03)
No	1,861 (99.09)	714,203 (99.97)
Thrombolysis		
Yes	1 (0.05)	182 (0.03)
No	1,877 (99.95)	714,215 (99.97)
Heart valve surgery		
Yes	108 (5.75)	211 (0.03)
No	1,770 (94.25)	714,186 (99.97)
**Medication**		
None	1,063 (56.60)	553,539 (77.48)
Antiplatelet agents		
Single Antiplatelets	328 (17.47)	139,498 (19.53)
Dual Antiplatelets	153 (8.15)	14,508 (2.03)
Anticoagulant		
Warfarin	181 (9.64)	1,999 (0.28)
Warfarin (Heparin bridge)	40 (2.13)	326 (0.05)
DOACs	113 (6.01)	4,726 (0.66)
**Dental procedure**		
Conservative treatment	39 (2.08)	54,448 (7.62)
Periodontal treatment	1,021 (54.37)	505,409 (70.74)
Tooth extraction	715 (38.07)	131,069 (18.35)
Implant related surgery	103 (5.48)	23,471 (3.28)

CVD, cardiovascular disease; CABG, Coronary artery bypass grafting

Values are n (%), as indicated.

In the single-institution dataset, postoperative bleeding was significantly associated with hypertension, diabetes mellitus, valve surgery, and antithrombotic use (Table 2). In the NHIS cohort, systemic bleeding and thromboembolic complications were significantly associated with sex, age, comorbidities, history of procedures, such as stent insertion or thrombolysis, antithrombotic use, and type of dental treatment (Table 3).

**Table 2:** Factors associated clinical characteristics influencing post-operative bleeding.

Characteristics	Post-operative bleeding		
	Not occurred (n = 1,837)	Occurred (n = 41)	*P*
**Sex**			
Male	824 (44.86)	17 (41.46)	.0536
Female	1,013 (55.14)	24 (58.54)	
**Age, mean±SD, year**	61.29 ± 12.01	64.34 ± 13.66	.1900
Comorbidities (Yes versus no)			
Hypertension	778 (42.35)	26 (63.41)	**.0017**⁎⁎
Diabetes mellitus	802 (43.66)	25 (60.98)	.0334*
Dyslipidemia	911 (49.59)	20 (48.78)	.5269
**CVD surgery (Yes versus no)**			
Coronary stenting	218 (11.87)	4 (9.76)	.6921
CABG	17 (0.93)	N/A	N/A
Thrombolysis	1 (0.05)	N/A	N/A
Heart valve surgery	101 (5.50)	7 (17.07)	.0338*
**Medication**			
Anticoagulant	317 (17.26)	17 (41.46)	**<.0001**⁎⁎⁎
Antiplatelet agents	465 (25.31)	16 (39.02)	
None	1,055 (57.43)	8 (19.51)	
**Drug discontinued (n=815)**			
Not discontinued	451 (56.52)	9 (52.94)	.1236
Discontinued	347 (43.48)	8 (42.06)	
**Dental treatment**			
Conservative treatment	39 (2.12)	N/A	.1579
Periodontal treatment	1,002 (54.55)	19 (46.34)	
Tooth extraction	697 (37.94)	18 (43.90)	
Dental implant surgery	99 (5.39)	4 (9.76)	
**Number of teeth treated**			
One tooth	507 (27.60)	6 (14.63)	.3947
Two teeth	236 (12.86)	6 (14.63)	
Three teeth	64 (3.48)	2 (4.88)	
Four or more teeth	1,030 (56.07)	27 (65.85)	

CVD, cardiovascular disease; CABG, Coronary artery bypass grafting

Values are n (%), as indicated.

⁎ p<0.05;

⁎⁎ p<0.01,

⁎⁎⁎ p<0.001

**Table 3:** Factors associated clinical characteristics influencing systemic complication.

Characteristics		Systemic bleeding			Thromboembolic complication		
		Not occurred (n = 712,269**)**	Occurred (n = 2,128)	*P*	Not occurred (n = 713,935)	Occurred (n = 462)	*P*
**Sex**							
	Male	370,744 (52.05)	1,025 (48.17)	**.0003**⁎⁎	371,512 (52.04)	257 (55.63)	.1245
	Female	341,525 (47.95)	1,103 (51.83)		342,423 (47.96)	205 (44.37)	
**Age, mean±SD, year**		60.58±11.44	65.05±11.74	**<.0001**⁎⁎⁎	60.58±11.44	69.01±11.87	**<.0001**⁎⁎⁎
**Comorbidities (Yes versus no)**							
	Hypertension	412,723 (57.94)	1,491 (70.07)	**<.0001**⁎⁎⁎	413,871 (57.97)	343 (74.24)	**<.0001**⁎⁎⁎
	Diabetes mellitus	277,247 (38.92)	1,058 (49.72)	**<.0001**⁎⁎⁎	278,087 (38.95)	218 (47.19)	**.0003**⁎⁎
	Dyslipidemia	466,288 (65.47)	1,480 (69.55)	**<.0001**⁎⁎⁎	467,439 (65.47)	329 (71.21)	**.0095**⁎⁎
**CVD surgery (Yes versus no)**							
	Stent insertion	5,598 (0.79)	34 (1.60)	**<.0001**⁎⁎⁎	5,619 (0.79)	13 (2.18)	**.0001**⁎⁎
	CABG	193 (0.03)	1 (0.05)	.4394	194 (0.03)	N/A	N/A
	Thrombolysis	176 (0.02)	6 (0.28)	**<.0001**⁎⁎⁎	175 (0.02)	7 (1.52)	**<.0001**⁎⁎⁎
	Heart valve surgery	209 (0.03)	2 (0.09)	.1312	210 (0.03)	1 (0.22)	.1276
**Medication**							
	Anticoagulant	6,795 (0.95)	57 (2.68)	**<.0001**⁎⁎⁎	6,831 (0.96)	21 (4.55)	**<.0001**⁎⁎⁎
	Antiplatelet agents	153,382 (21.53)	624 (29.32)		153,791 (21.54)	215 (46.54)	
	None	552,092 (77.51)	1,447 (68.00)		553,313 (77.50)	226 (48.92)	
**Dental treatment**							
	Conservative treatment	54,258 (7.62)	190 (8.90)	**<.0001**⁎⁎⁎	54,414 (7.62)	34 (7.36)	**<.0001**⁎⁎⁎
	Periodontal treatment	504,066 (70.77)	1,343 (63.11)		505,145 (70.76)	264 (57.14)	
	Tooth extraction	130,557 (18.33)	512 (24.06)		130,921 (18.34)	148 (32.03)	
	Dental implant surgery	23,388 (3.28)	83 (3.90)		23,455 (3.29)	16 (3.46)	

CVD, cardiovascular disease; CABG, Coronary artery bypass grafting

Values are n (%), as indicated.

*p < .05;

⁎⁎ *p* < .01,

⁎⁎⁎ *p* < .001.

The relationship between drug discontinuation and postoperative bleeding, including detailed rates and discontinuation dates for VKAs, DOACs, single antiplatelet agents, and dual antiplatelet agents, is summarized in Supplemental Table 7.

**Fig. 2:**
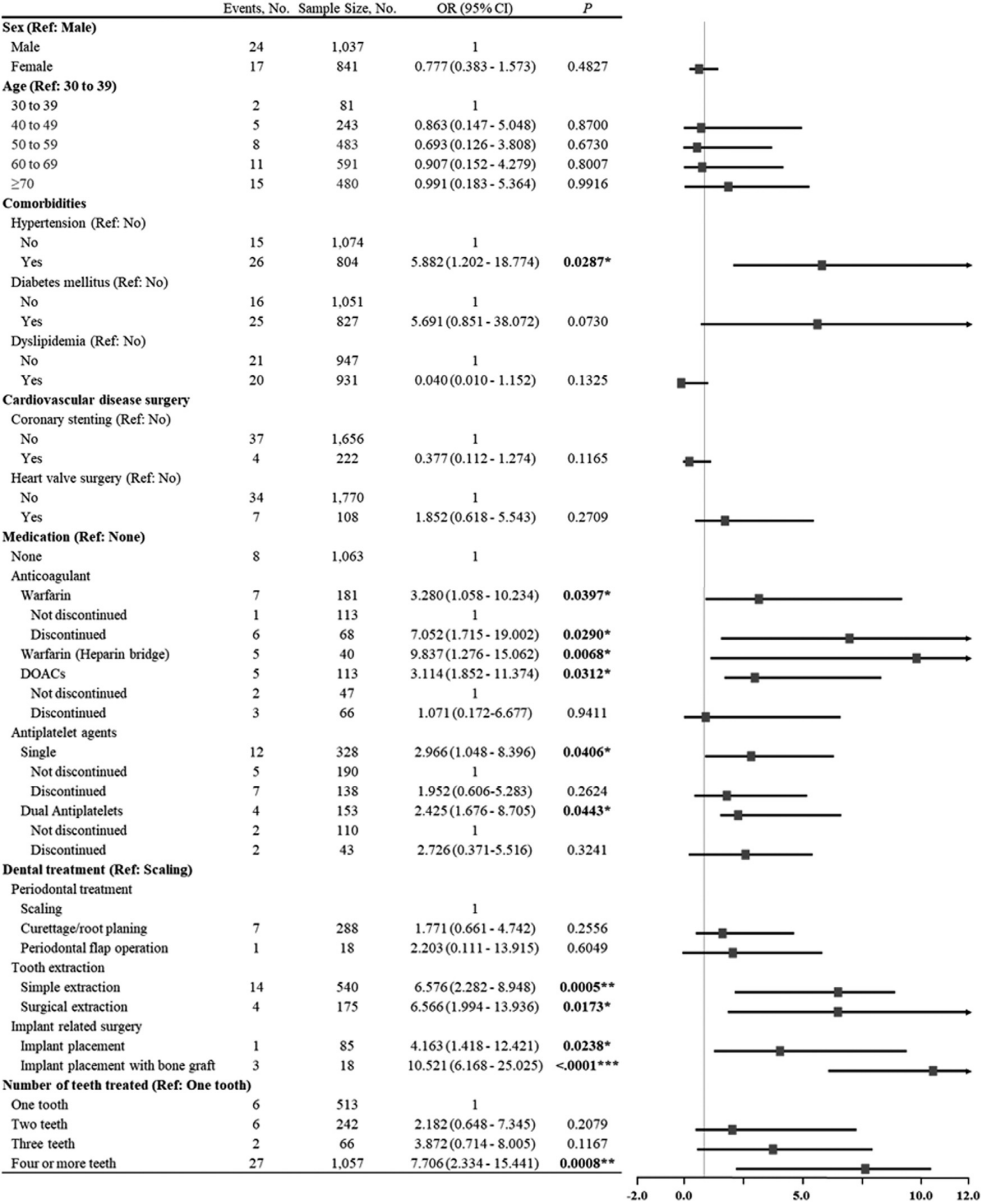
Multivariate logistic regression analysis of post-operative bleeding. Vertical line indicates no effect relative to the reference level. OR, odds ratio; CI, confidence interval. *p < .05; ^⁎⁎^*p* < .01, ^⁎⁎⁎^*p* < .001. Reference levels are as follows: Male (sex), 30 to 39 years (age), No comorbidities (hypertension, diabetes, dyslipidemia), No history of cardiovascular surgery (coronary stenting, heart valve surgery), No antithrombotic agent (medication), Scaling (dental treatment), 1 tooth treated (number of teeth treated).

Postoperative bleeding was associated with hypertension (odds ratio [OR, 5.882; 95% confidence interval [CI], 2.202-18.774), valve surgery (OR, 5.115; 95% CI, 1.299-17.621), and use of antithrombotic agents, especially VKAs with heparin bridging (OR, 9.837; 95% CI, 1.276-15.062) (Figure 2). Contrastingly, a history of stent insertion was associated with lower bleeding risk (OR, 0.368; 95% CI, 0.140-0.962). Simple and surgical extraction, implant placement, and implant placement with bone grafting were associated with a significantly increased risk of postoperative bleeding, with implant placement with bone grafting showing a high risk (OR, 10.521; 95% CI, 6.168-25.025). The treatment of ≥4 teeth increased the risk (OR, 7.706; 95% CI, 2.334-10.441).

**Fig. 3:**
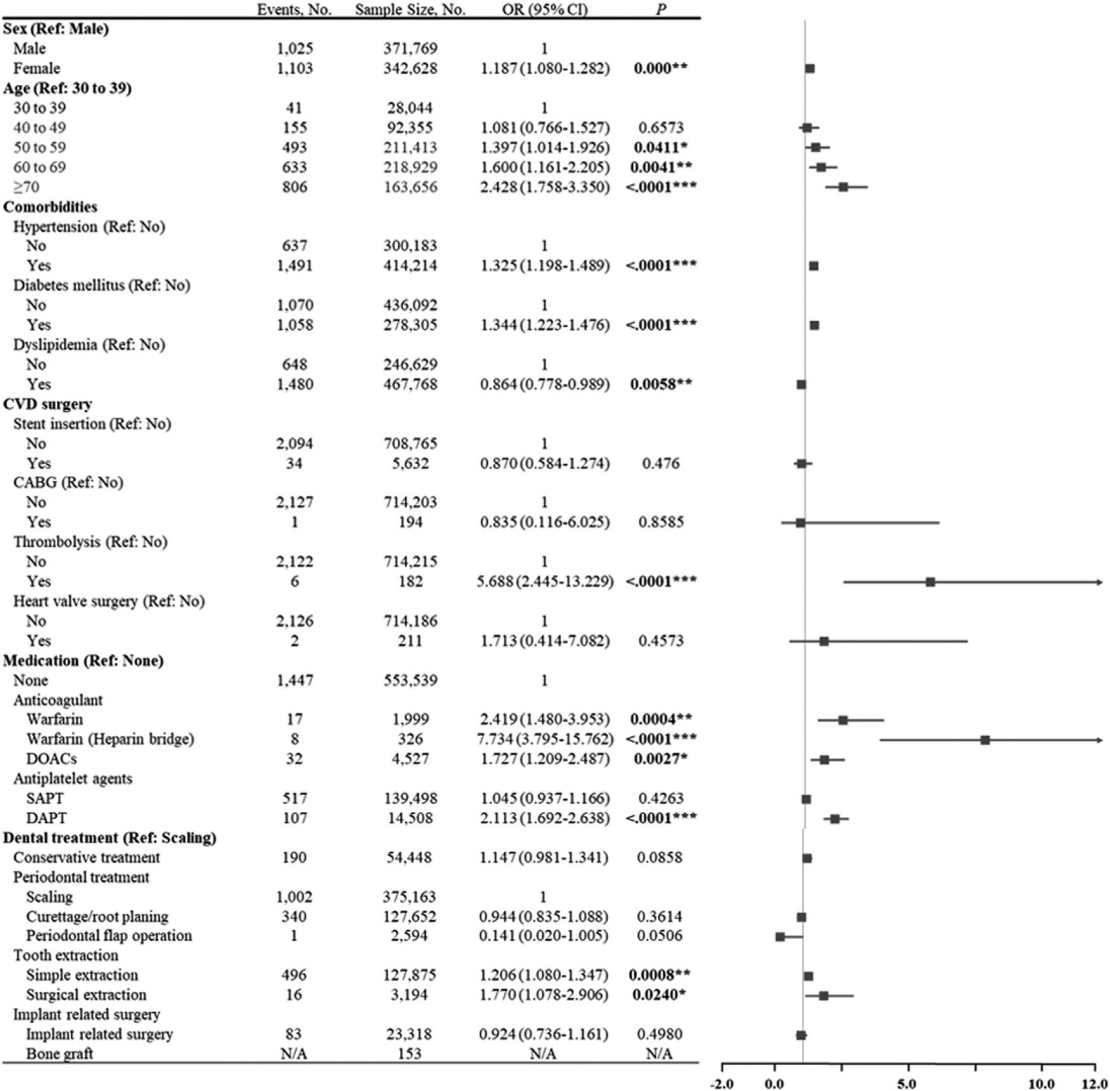
Multivariate logistic regression analysis of systemic bleeding. Vertical line indicates no effect relative to the reference level. OR, odds ratio; CI, confidence interval. *p < .05; ^⁎⁎^*p* < .01, ^⁎⁎⁎^*p* < .001. Reference levels are as follows: Male (sex), 30 to 39 years (age), No comorbidities (hypertension, diabetes, dyslipidemia), No history of cardiovascular surgery (coronary stenting, heart valve surgery), No antithrombotic agent (medication), Scaling (dental treatment), 1 tooth treated (number of teeth treated).

**Fig. 4:**
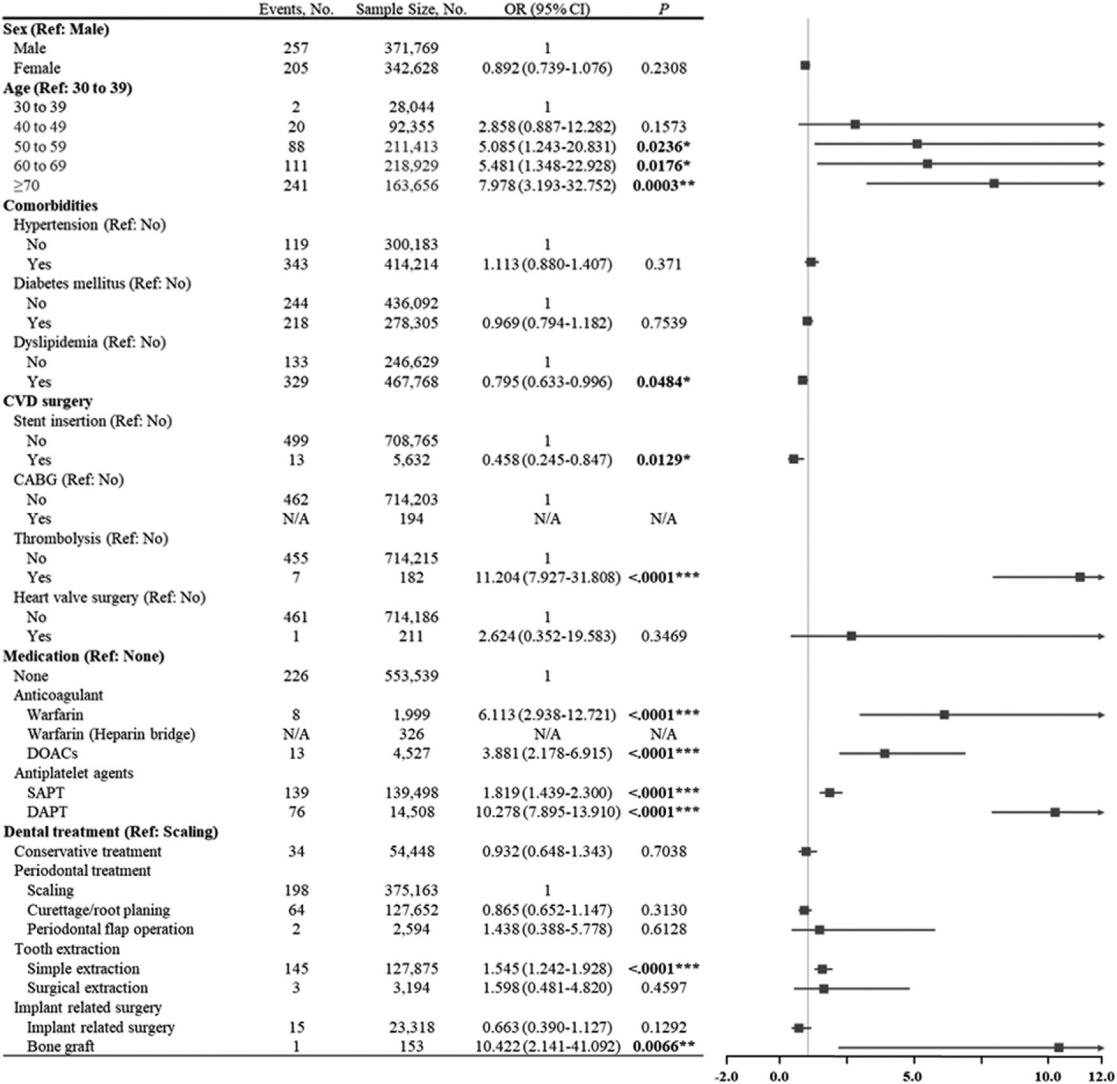
Multivariate logistic regression analysis of thromboembolic complications. Vertical line indicates no effect relative to the reference level. OR, odds ratio; CI, confidence interval. *p < .05; ^⁎⁎^*p* < .01, ^⁎⁎⁎^*p* < .001. Reference levels are as follows: Male (sex), 30 to 39 years (age), No comorbidities (hypertension, diabetes, dyslipidemia), No history of cardiovascular surgery (coronary stenting, heart valve surgery), No antithrombotic agent (medication), Scaling (dental treatment), 1 tooth treated (number of teeth treated).

Systemic bleeding and thromboembolic complications were significantly associated with females and older age (Fig. 3, Fig. 4). Hypertension (OR, 1.325; 95% CI, 1.198-1.489) and diabetes mellitus (OR, 1.344; 95% CI, 1.223-1.476) were associated with a higher risk of systemic bleeding. Thrombolysis (systemic bleeding OR, 5.688; 95% CI, 2.445-13.229; thromboembolic OR, 11.204; 95% CI, 7.927–31.808), DAPT (OR, 10.278; 95% CI, 7.895-13.910), and VKAs with heparin bridging (OR, 7.734; 95% CI, 3.795-15.762) were significantly associated with a higher risk of adverse outcomes. Simple (OR, 1.206; 95% CI, 1.080-1.347) and surgical extractions (OR, 1.770; 95% CI, 1.078–2.906) were associated with a higher risk of systemic bleeding, and bone grafting was associated with the highest observed risk of thromboembolic complications (OR, 10.422; 95% CI, 2.141-41.092).

## Discussion

This study evaluated factors associated with postoperative bleeding and systemic complications following dental procedures using SCRAP 2.0 and NHIS-NSC data. Antithrombotic therapy, particularly VKAs with heparin bridging, dental extraction, implant surgery with bone grafting, and hypertension, were significant risk factors.

### Anticoagulants: VKAs and DOACs

DOACs are widely used as alternatives to VKAs, especially in patients with nonvalvular atrial fibrillation. However, VKAs remain common in patients with contraindications for DOACs. This study found a significantly higher risk of postoperative bleeding in patients taking antithrombotic agents, consistent with those of previous studies that reported higher bleeding risks with VKAs than with antiplatelet agents.^21^ The NHIS-NSC data showed that VKA users were younger on average than DOAC users; however, VKAs were associated with higher systemic complication risks. Previous studies have reported that DOACs are more effective than VKAs in reducing major and intracranial bleeding.^22^^,^^23^

Heparin bridging was associated with an increased incidence of postoperative and systemic complications, consistent with previous studies reporting that discontinuing VKAs and bridging with heparin or enoxaparin increases thrombotic and bleeding events.^24^ For dental procedures with low bleeding risk, VKAs can be continued with local hemostatic measures or discontinued 2–3 days preoperatively if the INR is >3.5 and the bleeding risk is moderate.^25^ This study found no significant difference in dental or systemic complications between drug discontinuation and continuation of DOACs, consistent with other studies showing that the risk of bleeding is more influenced by comorbidities and complex dental procedures, such as implant placement.^26^^,^^27^

### Antiplatelet agents: SAPT and DAPT

Antiplatelet agents, such as aspirin and clopidogrel, are commonly prescribed as SAPT or DAPT. In this study, antiplatelets were prescribed to 22% to 26% of patients. The risk of postoperative bleeding was similarly high in patients receiving SAPT and DAPT compared with those not receiving antiplatelet therapy; however, there was no significant association with drug continuation/discontinuation. Previous reports have shown that postoperative bleeding did not significantly increase without drug discontinuation and that local hemostatic measures were effective.^28^

The NHIS-NSC data showed a higher risk of thromboembolic complications in patients receiving DAPT than in those receiving SAPT who were not taking antithrombotic agents. Brown et al. reported that short-term DAPT after transient ischemic attack or minor stroke reduces ischemic recurrence; however, long-term use increases major bleeding risks.^29^^,^^30^ However, this study excluded SAPT and DAPT maintenance periods, which may have affected the interpretation of results. Therefore, further research is needed to collect specific data on the duration of drug administration and timing of discontinuation.

### Dental procedures: extraction and implant-related factors

Previous studies reported post-extraction bleeding rates of 2–26% in patients receiving antithrombotic therapy.^31^ Previous studies reported that complicated extractions, extractions with bone grafting, and multiple implant placements were associated with higher bleeding risk.^32^^,^^33^ Extraction, implant surgery, and implant surgery with bone grafting were significantly associated with postoperative bleeding and systemic complications, particularly in more invasive dental procedures.

In the NHIS-NSC data, simple extraction was associated with a higher risk of systemic bleeding and thromboembolic complications, whereas surgical extraction was associated only with systemic bleeding. Simple extractions were defined as those performed without flap elevation. However, the classification based on procedure codes in the NHIS may have led to inaccuracies.

Previous studies reported that extracting up to three teeth was safe if the PT-INR was <3.5 in the absence of other bleeding risks.^31^^,^^34^ This study revealed no significant difference between the number of teeth treated and postoperative bleeding. However, because scaling with a low risk of bleeding involves the treatment of at least one-third of the jaw or all teeth, dental treatment variables require adjustment. Multivariate logistic regression analysis showed that the risk of postoperative bleeding increased if ≥4 teeth were treated.

### Comorbidities and CVD surgery

Previous studies reported history of stroke, older age, hypertension, and diabetes mellitus as independent risk factors for thromboembolism in atrial fibrillation.^35^ Similarly, hypertension and diabetes were risk factors for postoperative bleeding and systemic complications. These results indicate a need to consider patient comorbidities and procedural invasiveness during dental treatment planning.

Stent insertion was associated with a 0.458-fold lower risk of thromboembolic complications, likely because the outcome variable in this study was defined as thromboembolic complications. Conversely, major adverse cardiac events were defined as readmission for acute coronary syndrome or coronary revascularization and death.^36^ Noncardiac surgery can be performed safely at least six months after stent insertion.^37^ Although percutaneous coronary intervention (PCI) patients have higher bleeding risks with triple antithrombotic therapy,^38^ such cases were excluded from this analysis. The lower OR of thromboembolic complications in patients with stent implantation may be because of the fact that most noncardiac surgeries were performed in the stable period after stent implantation.

Thrombolysis, which is used to treat acute ischemic events, is associated with a 5 to 11-fold increased risk of systemic complications. Previous research has reported higher mortality and bleeding rates among patients undergoing thrombolysis than among those undergoing PCI.^39^ Therefore, careful attention may be required for bleeding management during dental treatment, depending on the patient’s cardiovascular-related clinical characteristics and medications.

This large-scale study used the Severance Clinical Research Analysis Portal (SCRAP 2.0) and NHIS-NSC to examine a large number of patients receiving anticoagulants, antiplatelet agents, and various dental treatments. The SCRAP 2.0 provided detailed clinical information, including treatment history and medication discontinuation. The NHIS-NSC data offered population-based generalizability across multiple hospitals, but lacked detailed clinical information and relied on reimbursement codes.

Systemic complications were identified using administrative codes that may not fully reflect clinical status or severity. This limitation may lead to inaccurate estimation of outcomes, particularly due to coding inaccuracies or incomplete capture of events, and should be considered when interpreting the findings. Furthermore, implant procedures identified in the NHIS dataset are limited to patients aged ≥65 years due to insurance coverage policies. Therefore, implant-related outcomes may not be generalizable to younger populations, and this age restriction may have influenced the estimated risks associated with implant procedures.

This study focused on individual anticoagulants and antiplatelet agents but excluded VKA triple therapy, which has been associated with a 3-fold higher risk of major bleeding compared with VKA monotherapy.^20^ The lack of investigation into dental disease is a limitation, considering that periodontal inflammation may increase the risk of bleeding in anticoagulant users.^40^ Additionally, NHIS-NSC data are limited to implants provided to patients aged ≥65 years, as it includes only implants covered by the NHI. This may be due to a selection bias in implant-related outcomes. To reduce confounding factors, patients with terminal illness, organ dysfunction, or multiple procedures within two weeks were excluded to ensure that postoperative complications were attributable to single interventions. These criteria enhance the validity of the study but may limit its generalizability.

## Conclusions

Patients receiving VKAs with heparin bridging and those undergoing implant surgery with bone grafting had the highest observed risks of postoperative bleeding and systemic complications following dental procedures. In contrast, the relatively low risk associated with single antiplatelet therapy and implant placement alone may inform risk stratification and procedural planning for dental treatment in patients receiving antithrombotic therapy.

## Data availability

The data that support the findings of this study are available from Korean National Health Insurance Service. Restrictions apply to the availability of these data, which were used under license for this study. Data are available from https://nhiss.nhis.or.kr with the permission of Korean National Health Insurance Service.

## Declaration of competing interest

The authors declare that they have no competing interests.

## Appendix Supplementary materials

Image, application 1

## Footnotes

Supplementary material associated with this article can be found in the online version at doi:10.1016/j.identj.2026.109649.
