# Supplementary material for: Dental Complications in Antithrombotic Patients: Evidence From a Nationwide Cohort and a Single-Institution Dataset
Source: Int Dent J. 2026 Jun 1;76(4):109649. doi: 10.1016/j.identj.2026.109649 (PMC13251685; doi:10.1016/j.identj.2026.109649)
Supplement: Supplementary file 1 [file mmc1.pdf]

Supplemental Table 1. Codes and definitions of CVD characteristics

	Code	Name of Diagnosis
Heart Valve Disease (HVD)	I05	Rheumatic mitral valve diseases
	I06	Rheumatic aortic valve diseases
	I07	Rheumatic tricuspid valve diseases
	I08	Multiple valve diseases
	I34	Mitral (valve) insufficiency
	I35	Nonrheumatic aortic valve disorders
	I36	Nonrheumatic tricuspid valve disorders
	I37	Pulmonary valve disorders
Ischemic Heart Disease (IHD)	I21	Acute myocardial infarction
	I22	Subsequent myocardial infarction
	I23	Certain current complications following acute myocardial infarction
	I24	Other acute ischaemic heart diseases
	I25	Chronic ischaemic heart disease
Stroke	I60	Subarachnoid haemorrhage
	I61	Intracerebral haemorrhage
	I63	Cerebral infarction
	I64	Stroke, not specified as haemorrhage or infarction
	I69	Sequelae of cerebrovascular disease
	G45.8	Other transient cerebral ischaemic attacks and related syndromes
	G45.9	Transient cerebral ischaemic attack, unspecified
Peripheral Artery Disease (PAD)	I71	Aortic aneurysm and dissection
	I74	Arterial embolism and thrombosis
Atrial Fibrillation (AF)	I48	Atrial fibrillation and flutter
Heart Failure (HF)	I50	Heart failure
	I11.0	Hypertensive heart disease with (congestive) heart failure
	I13.0	Hypertensive heart and renal disease with (congestive) heart failure
	I13.2	Hypertensive heart and renal disease with both (congestive) heart failure and renal failure

CVD, cardiovascular disease; ICD-10, International Standard Classification of Diseases, 10th Revision

Supplemental Table 2. Codes and definitions of outcomes

	Code	Name of Diagnosis
Hemorrhage from the respiratory passages	R04	Haemorrhage from respiratory passages
Hemothorax or hematuria	N02	Recurrent and persistent haematuria
	R31	Unspecified haematuria
	J94.2	Haemothorax
Hemorrhagic digestive system disorders and ulcers	K25	Gastric ulcer
	K26	Duodenal ulcer
	K27	Peptic ulcer, site unspecified
	K28	Gastrojejunal ulcer
	K92	Other diseases of digestive system
Anemia	D50	Iron deficiency anaemia
	D62	Acute posthaemorrhagic anaemia
Intracranial hemorrhage or injury	I60	Subarachnoid haemorrhage
	I61	Intracerebral haemorrhage
	I62	Other nontraumatic intracranial haemorrhage
	S06	Intracranial injury
**Thromboembolic complications**	**Code**	**Name of Diagnosis**
Stroke	I63	Cerebral infarction
	I64	Stroke, not specified as haemorrhage or infarction
	I69	Sequelae of cerebrovascular disease
	G45.8	Other transient cerebral ischaemic attacks and related syndromes
	G48.9	Transient cerebral ischaemic attack, unspecified
Arterial embolism and thrombosis	I74	Arterial embolism and thrombosis
Complications after acute myocardial infarction	I23	Certain current complications following acute myocardial infarction

CVD, cardiovascular disease; ICD-10, International Standard Classification of Diseases, 10th Revision

Supplemental Table 3. Comparison of characteristics according to antithrombotic agents in patients diagnosed with CVD (Single-institution)

Characteristics	None	Anticoagulant			Antiplatelet agents	
		Warfarin	Warfarin (heparin bridge)	DOACs	SAPT	DAPT
**Total**	1,063 (56.60)	181 (9.64)	40 (2.13)	113 (6.02)	328 (17.47)	153 (8.15)
**Sex**						
Male	532 (50.05)	108 (59.67)	19 (47.50)	59 (52.21)	196 (59.76)	123 (80.39)
Female	531 (49.95)	73 (40.33)	21 (52.50)	54 (47.79)	132 (40.24)	30 (19.61)
**Age, mean±SD, year**	59.64±12.12	59.76±12.43	60.00±13.30	66.55±10.49	65.38±10.81	63.12±11.19
**Comorbidities (Yes versus no)**						
Hypertension	369 (34.71)	96 (53.04)	16 (40.00)	69 (61.06)	173 (52.74)	81 (52.94)
Diabetes mellitus	352 (33.11)	104 (57.46)	19 (47.50)	74 (65.49)	181 (55.18)	97 (63.40)
Dyslipidemia	435 (40.92)	105 (58.01)	19 (47.50)	76 (67.26)	193 (58.84)	103 (67.32)
**CVD surgery (Yes versus no)**						
Stent insertion	19 (8.56)	1 (0.45)	4 (1.80)	5 (2.25)	102 (45.95)	91 (40.99)
CABG	3 (17.65)	1 (5.88)	1 (5.88)	N/A	7 (41.18)	5 (29.41)
Thrombolysis	1 (100)	N/A	N/A	N/A	N/A	N/A
Heart valve surgery	5 (4.63)	75 (69.44)	6 (5.56)	6 (5.56)	9 (8.33)	7 (6.48)

CABG, coronary artery bypass grafting; CVD, cardiovascular disease; DOACs, direct oral anticoagulants; DAPT, dual antiplatelet therapy; SAPT, single anti platelet therapy.

Supplemental Table 4. Comparison of characteristics according to dental treatment in patients diagnosed with CVD (Single-institution)

Characteristics	Conservative treatment	Periodontal treatment			Tooth extraction		Implant-related surgery
		Scaling	Curettage / Root planing	Periodontal flap operation	Simple extraction	Surgical extraction	
**Total**	39 (2.08)	715 (38.07)	288 (15.34)	18 (0.98)	540 (28.75)	175 (9.32)	103 (5.48)
**Sex**							
Male	21 (53.85)	391 (54.69)	154 (53.47)	7 (38.89)	307 (56.85)	98 (56.00)	59 (57.28)
Female	18 (46.15)	324 (45.31)	134 (46.53)	11 (61.11)	233 (43.15)	77 (44.00)	44 (42.72)
**Age, mean±SD, year**	59.10±13.09	59.79±11.84	59.54±10.97	66.44±15.88	64.12±11.85	59.97±13.91	65.17±9.24
**Comorbidities (Yes versus no)**							
Hypertension	19 (48.72)	298 (41.68)	127 (44.10)	10 (55.56)	216 (40.00)	79 (45.14)	55 (53.40)
Diabetes mellitus	20 (51.28)	292 (40.84)	135 (46.88)	8 (44.44)	243 (45.00)	76 (43.43)	53 (51.46)
Dyslipidemia	21 (53.85)	335 (46.85)	145 (50.35)	10 (55.56)	276 (51.11)	83 (47.43)	61 (59.22)
**CVD surgery (Yes versus no)**							
Stent insertion	4 (1.80)	35 (15.77)	21 (9.46)	2 (0.90)	107 (48.20)	24 (10.81)	29 (13.06)
CABG	N/A	8 (47.06)	2 (11.76)	N/A	5 (29.41)	1 (5.88)	1 (5.88)
Thrombolysis	N/A	N/A	N/A	N/A	N/A	1 (100)	N/A
Heart valve surgery	3 (2.78)	47 (43.52)	28 (25.93)	1 (0.93)	17 (15.74)	6 (3.43)	6 (5.56)

CABG, coronary artery bypass grafting; CVD, cardiovascular disease.

Supplemental Table 5. Comparison of characteristics according to antithrombotic agents in patients diagnosed with CVD (NHIS-NSC)

Characteristics	None	Anticoagulant			Antiplatelet agents	
		Warfarin	Warfarin (heparin bridge)	DOACs	SAPT	DAPT
**Total**	553,539 (77.48)	1,999 (0.28)	326 (0.05)	4,527 (0.63)	139,498 (19.53)	14,508 (2.03)
**Sex**						
Male	279,140 (50.43)	1,097 (54.88)	210 (64.42)	2,688 (59.38)	78,365 (56.18)	10,269 (70.78)
Female	274,399 (49.57)	902 (45.12)	116 (35.58)	1,839 (40.62)	61,133 (43.82)	4,239 (29.22)
**Age, mean±SD, year**	58.78±11.27	66.10±10.90	63.78±9.69	71.88±8.43	66.65±9.66	66.98±10.09
**Comorbidities (Yes versus no)**						
Hypertension	272,558 (49.24)	1,581 (79.09)	279 (85.58)	4,204 (92.87)	122,482 (87.80)	13,110 (90.36)
Diabetes mellitus	182,880 (33.04)	813 (40.67)	186 (57.06)	2,588 (57.17)	82,737 (59.31)	9101 (62.73)
Dyslipidemia	328,066 (59.27)	1,526 (76.34)	294 (90.18)	3,868 (85.44)	120,201 (86.17)	13,813 (95.21)
**CVD surgery (Yes versus no)**						
Stent insertion	165 (2.93)	8 (0.14)	70 (1.24)	37 (0.66)	1,786 (31.71)	3,566 (63.32)
CABG	15 (7.73)	8 (4.12)	2 (1.03)	N/A	58 (29.90)	111 (57.22)
Thrombolysis	13 (7.14)	13 (7.14)	4 (2.20)	27 (14.84)	32 (17.58)	93 (51.10)
Heart valve surgery	27 (12.80)	91 (43.13)	1 (0.47)	9 (4.27)	72 (34.12)	11 (5.21)

CABG, coronary artery bypass grafting; CVD, cardiovascular disease; DOACs, direct oral anticoagulants; DAPT, dual antiplatelet therapy; SAPT, single anti platelet therapy.

Supplemental Table 6. Comparison of characteristics according to dental treatment in patients diagnosed with CVD (NHIS-NSC)

Characteristics	Conservative treatment	Periodontal treatment			Tooth extraction		Implant-related surgery	
		Scaling	Curettage / Root planing	Periodontal flap operation	Simple extraction	Surgical extraction	Implant surgery	Bone graft
**Total**	54,448 (7.62)	375,163 (52.51)	127,652 (17.87)	2,594 (0.36)	127,875 (17.90)	3,194 (0.45)	23,318 (3.26)	153 (0.02)
**Sex**								
Male	28,753 (52.81)	189,934 (50.63)	67,014 (52.50)	1,417 (54.63)	71,062 (55.57)	1,891 (59.20)	11,615 (49.81)	83 (54.25)
Female	25,695 (47.19)	185,229 (49.37)	60,638 (47.50)	1,177 (45.37)	56,813 (44.43)	1,303 (40.80)	11,703 (50.19)	70 (45.75)
**Age, mean±SD, year**	62.53±11.32	58.55±11.22	60.26±10.59	59.34±9.69	64.24±11.56	58.90±14.48	71.09±6.10	57.22±8.78
**Comorbidities (Yes versus no)**								
Hypertension	32,920 (60.46)	203,620 (54.28)	75,203 (58.91)	1,534 (59.14)	82,169 (64.26)	1,851 (57.95)	16,846 (72.24)	71 (46.41)
Diabetes mellitus	22,393 (41.13)	135,091 (36.01)	50,896 (39.87)	1,021 (39.36)	56,261 (44.00)	1,197 (37.48)	11,386 (48.83)	60 (39.22)
Dyslipidemia	82,341 (64.39)	243,589 (64.93)	85,494 (66.97)	1,703 (65.65)	82,341 (64.39)	1,975 (61.83)	16,918 (72.55)	94 (61.44)
**CVD surgery (Yes versus no)**								
Stent insertion	552 (9.80)	2,559 (45.44)	971 (17.24)	15 (0.27)	1,213 (21.54)	31 (0.55)	291 (5.17)	N/A
CABG	16 (8.25)	94 (48.45)	36 (18.56)	N/A	40 (20.62)	2 (1.03)	6 (3.09)	N/A
Thrombolysis	15 (8.24)	88 (48.35)	33 (18.13)	N/A	40 (21.98)	1 (0.55)	5 (2.75)	N/A
Heart valve surgery	9 (4.27)	107 (50.71)	36 (17.06)	2 (0.95)	48 (22.75)	1 (0.47)	8 (3.79)	N/A

CABG, coronary artery bypass grafting; CVD, cardiovascular disease.

Supplemental Table 7. Factors affecting postoperative bleeding according to discontinuation and discontinuation date based on medication

Drug discontinued		Postoperative bleeding		
		Not occurred	Occurred	*P*
Anticoaugulant	Warfarin (n=181)			
	Continued	62 (35.63)	4 (57.14)	0.1960
	Discontinued	112 (64.37)	3 (42.86)	
	Discontinuation date, mean ± SD, day	3.35 ± 1.94	3.75 ± 2.22	0.5643
	DOACs (n=113)			
	Continued	63 (58.33)	3 (60.00)	0.9411
	Discontinued	45 (41.67)	2 (40.00)	
	Discontinuation date, mean ± SD, day	2.33 ± 1.53	2.22 ± 1.97	0.9164
Antiplatelet agents	SAPT (n=328)			
	Continued	132 (41.77)	8 (66.67)	0.0870
	Discontinued	184 (58.23)	4 (33.33)	
	Discontinuation date, mean ± SD, day	5.12 ± 2.18	4.67 ± 1.51	0.5103
	DAPT (n=153)			
	Continued	40 (26.85)	3 (75.00)	0.3058
	Discontinued	109 (73.15)	1 (25.00)	
	Discontinuation date, mean ± SD, day	4.80 ± 1.49	4.67 ± 0.58	0.8520

DOACs, direct oral anticoagulants; DAPT, dual antiplatelet therapy; SAPT, single antiplatelet therapy.
